# A Case of an Inflammatory Myofibroblastic Tumor Mimicking Appendicitis

**DOI:** 10.7759/cureus.14059

**Published:** 2021-03-23

**Authors:** Rene Flores, Henriette De La Garza, Alfredo A Santillan-Gomez

**Affiliations:** 1 Surgery, Beth Israel Deaconess Medical Center, Harvard Medical School, Boston, USA; 2 Surgical Oncology, Methodist Health System, San Antonio, USA; 3 Dermatology, Boston University School of Medicine, Boston, USA

**Keywords:** inflammatory myofibroblastic tumor, appendicitis, ascending colon, imt

## Abstract

Inflammatory myofibroblastic tumors (IMTs) are rare tumors that have been described only in a few cases in the literature. IMTs are mesenchymal neoplasms that typically affect children and young adults. The most common anatomical locations are the abdominopelvic region, lung, and retroperitoneum, but any site may be involved. Given that there are no clinical or radiographic characteristics specific to IMTs, the diagnosis is made by pathology. We report on a young woman presenting with an acute appendicitis-like clinical picture due to an IMT located in the ascending colon to raise awareness of this rare, but possible presentation.

## Introduction

Inflammatory myofibroblastic tumors (IMTs) are uncommon tumors with an unclear etiology that can present at any age with a varied range of clinical presentations. The true incidence and prevalence of IMTs are difficult to estimate as the definition and nomenclature of fibroinflammatory conditions are still evolving [1]. The most common site of presentation is the lungs, which account for a third of presenting cases, with the remaining two-thirds being in the abdominopelvic region [2]. The majority of IMTs present with mass effects, causing a delay in diagnosis due to many possible differential diagnoses.

## Case presentation

A 20-year-old female presented to the emergency department with right lower quadrant abdominal pain that was initiated 24 hours before arrival. The pain was of sudden onset, sharp, and colicky with progressing intensity. The patient denied any nausea, vomiting, gastrointestinal or urinary symptoms. Her past medical history and laboratory results including urinalysis and beta-human chorionic gonadotropin (B-HCG) were unremarkable, except for a slightly elevated white blood cell count (12,300/μl), C-reactive protein level of 2.87 mg/dl, and a temperature of 100.5°F (38°C). 

**Figure 1:**
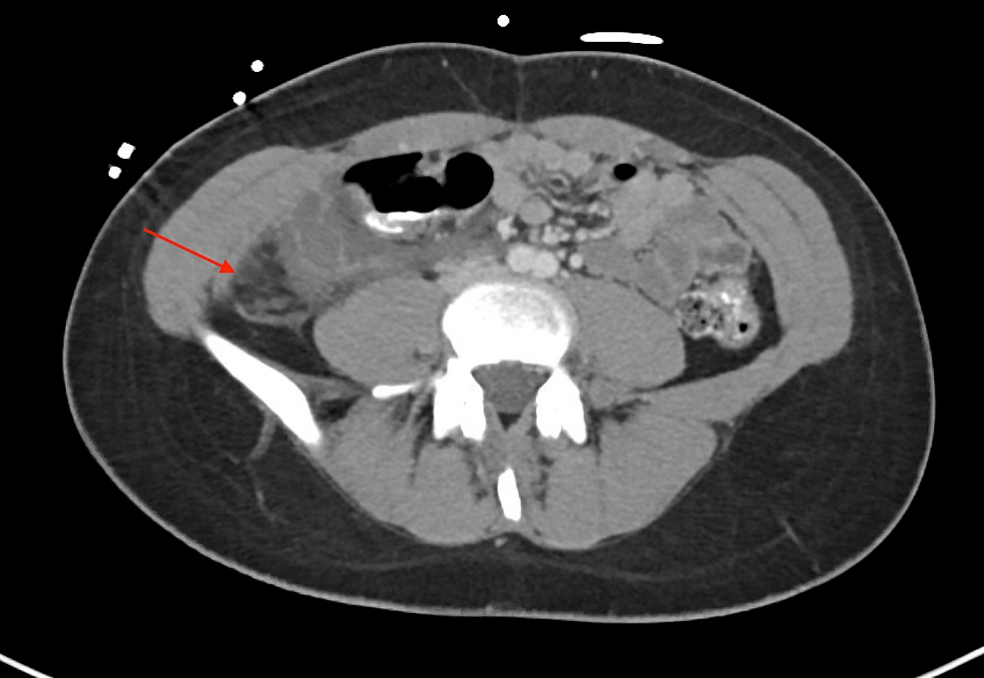
Abdominal CT scan with IV contrast revealing an inflammatory process involving the cecum and ascending colon.

Physical examination revealed a soft abdomen with tenderness in the right lower quadrant on palpation and a positive McBurney sign with signs of peritoneal irritation. A CT showed an extensive inflammatory process around the cecum and the ascending colon with possible ruptured mucocele of the appendix (Figure 1).

**Figure 2:**
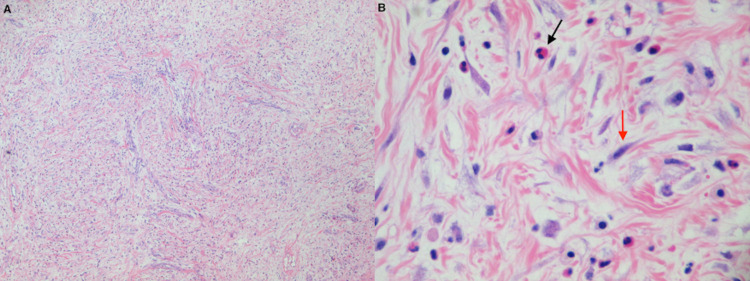
Microscopic appearance of IMT. A. Low power (×40) H&E staining demonstrating a mild fibroblastic background and collagenous stroma with inflammatory cells consisting of lymphocytes, plasma cells, and scattered eosinophils and neutrophils. B. High power (x400) microscopic view of H&E stain demonstrating predominance of spindle cells (red arrow) and inflammatory cells (black arrow). IMT: inflammatory myofibroblastic tumor; H&E: hematoxylin & eosin

An exploratory laparotomy was performed where a normal-appearing appendix was found with no free fluid in the abdominal cavity. The uterus, fallopian tubes, and ovaries were also normal. There was a mass of approximately 5 cm attached to the ascending colon near the hepatic flexure, located from the subepithelial to subserosal planes, mostly intraluminal. A right hemicolectomy was performed and sent to pathology who reported a tan-yellow firm mass that measured 6.0 x 3.0 cm. The mass was composed of a mild fibroblastic background with inflammatory cells consisting of lymphocytes, plasma cells, numerous eosinophils, and scattered neutrophils. The histological examination also revealed spindle cells with enlarged myofibroblastic-type nuclei with a vascular pattern without any considerable nuclear atypia or mitotic activity. The immunohistochemical studies showed that tumor cells were positive for desmin and actin and negative for anaplastic lymphoma kinase (ALK1) protein, cluster of differentiation 34 (CD34), c-kit, and Epstein-Barr virus (EBV) (Figure 2). Based on the above clinical and histopathologic findings, the patient received a diagnosis of IMT. The patient had an uneventful postoperative course, with complete resolution of her symptoms and laboratory values after the surgery. No evidence of recurrence has been noticed in the two-year follow-up and she remains under clinical surveillance.

## Discussion

IMT is defined as a lesion composed of myofibroblastic spindle cells accompanied by an inflammatory infiltrate of plasma cells, lymphocytes, and eosinophils. It occurs primarily in the soft tissue and viscera of children and young adults. The IMTs have been reported in many locations including lung, liver, spleen, stomach, esophagus, abdominal cavity, omentum, retroperitoneum, orbits, spinal meninges, heart, thyroid gland, and kidney [2].

To date, the etiologic factors responsible for these tumors remain unclear. Numerous implications and associations have surfaced, yet none has revealed the pathogenic mechanism of the condition. IMTs may represent an immunologic response to an infectious agent, Campylobacter jejuni, Epstein-Barr virus, and Escherichia coli have been associated with these tumors. Trauma, steroid use, abdominal surgery, and genetic factors have also been reported [3]. On the other hand, molecular studies have demonstrated rearrangements in chromosome 2p33 in the ALK gene in up to 67% of IMT cases, supporting a neoplastic origin [4].

The diagnosis of IMTs may be delayed owing to nonspecific presenting symptoms. Patients generally present with mass or site-specific symptoms including vague abdominal pain or gastrointestinal complaints in intraabdominal lesions, and cough, chest pain, or, rarely, hemoptysis in pulmonary tumors. Patients may also present with fever, weight loss, malaise, anemia, thrombocytosis, and leukocytosis. [5,6]. Colorectal IMT’s are uncommon, and less than thirty cases have been reported in the literature, where the descending colon and sigmoid colon seem to be the most frequently involved sites [7]. Rarely, the presentation may be complicated by intestinal obstruction, intussusception, or acute abdomen mimicking acute appendicitis.

The imaging features of IMTs are multifaceted and are likely related to inflammatory cell infiltration and the degree of stromal fibrosis. Imaging appearances vary from an ill-defined, infiltrating lesion to a well-circumscribed, soft tissue mass. Variable attenuation and echogenicity are noted at CT and ultrasonography, respectively [1]. These lesions are diagnosed as masses relating to their anatomic location and are impossible to differentiate from a malignant process based solely on radiographic studies. Given that there are no clinical or radiographic characteristics specific to IMTs, the diagnosis is made by pathology. Grossly, IMTs have a white or tan surface and be either firm, gelatinous or fleshy. Tumor size range from 1 cm to more than 20 cm, although the mean size is 6 cm. Histologically, they are characterized by cellular spindle cell proliferation in a myxoid to collagenous stroma with a prominent inflammatory infiltrate composed primarily of plasma cells and lymphocytes, with occasional admixed eosinophils and neutrophils. The mitotic rate is low. By immunohistochemistry, approximately 71% of IMTs are positive for ALK [3]. Whilst ALK positivity is helpful if present, its absence does not rule out the diagnosis. The pathogenesis of IMTs lacking ALK expression is not clear. However, in a recent study using next-generation sequencing, six of nine ALK-negative IMT tumors showed the presence of fusions involving ROS-1 or platelet-derived growth factor receptor beta (PDGFRb) genes, suggesting that IMT is largely a kinase fusion-driven neoplasm [8]. The IMTs may also be positive for vimentin, actin, desmin, and cytokeratin [9]. There is no consensus about reliable pathologic predictors of the biology of IMTs, although the presence of ganglion-like cells, p53 expression, and aneuploidy has been associated with more aggressive behavior [1]. The differential diagnosis includes schwannomas, desmoid tumors, gastrointestinal stromal tumors (GIST), inflammatory fibroid polyps, leiomyomas, and leiomyosarcomas [10].

The IMTs generally have a benign course, and recurrence or metastasis is rare. The treatment of choice is surgical excision, which resolves the mass effects and the systemic manifestations [9]. Chemotherapy, radiotherapy, nonsteroidal anti-inflammatory drugs (NSAIDs), and steroids have been used as alternative treatment modalities [10].

## Conclusions

The differential diagnosis of IMT may be wide and difficult even at the microscopic level. The most critical ambiguity is the etiology behind this broadly presenting condition. We report this case to raise awareness of this rare, but possible presentation of IMTs. The current level of evidence on IMTs is limited and based solely on anecdotal studies, case reports, and case series. Further research is needed to better understand the unpredictable nature of these tumors.
